# Draft Genome Sequence of *Tannerella forsythia* Type Strain ATCC 43037

**DOI:** 10.1128/genomeA.00660-15

**Published:** 2015-06-11

**Authors:** Valentin Friedrich, Stephan Pabinger, Tsute Chen, Paul Messner, Floyd E. Dewhirst, Christina Schäffer

**Affiliations:** a Department of NanoBiotechnology, NanoGlycobiology Unit, Universität für Bodenkultur Wien, Vienna, Austria; b AIT Austrian Institute of Technology, Health and Environment Department, Molecular Diagnostics, Vienna, Austria; c Department of Microbiology, The Forsyth Institute, Cambridge, Massachusetts, USA; d Department of Oral Medicine, Infection and Immunity, Harvard School of Dental Medicine, Boston, Massachusetts, USA

## Abstract

*Tannerella forsythia* is an oral pathogen implicated in the development of periodontitis. Here, we report the draft genome sequence of the *Tannerella forsythia* strain ATCC 43037. The previously available genome of this designation (NCBI reference sequence NC_016610.1) was discovered to be derived from a different strain, FDC 92A2 (= ATCC BAA-2717).

## GENOME ANNOUNCEMENT

*Tannerella forsythia* (formerly *Bacteroides forsythus*) is an anaerobic Gram-negative oral pathogen that was originally isolated from the human oral cavity of periodontal disease patients. Together with *Porphyromonas gingivalis* and *Treponema denticola*, it constitutes the “red complex” consortium that inhabits the dental plaque biofilms (1) and is strongly associated with the pathogenesis of severe and chronic periodontitis. Furthermore, there is mounting evidence for a link between periodontitis and a range of systemic medical conditions, including cardiovascular diseases, diabetes, obesity and rheumatoid arthritis (2). Despite its identification as a major periodontal pathogen, *T. forsythia* is an underinvestigated organism, especially with regard to its virulence factors (3, 4). This is mainly due to the bacterium’s fastidious growth and recalcitrance to genetic manipulation (5). To advance the future understanding of the virulence mechanisms of *T. forsythia* at the molecular level, knowledge of the underlying genome sequence is indispensable.

The type strain of *T. forsythia* (ATCC 43037 = FDC 338) was isolated by Tanner et al. and deposited with ATCC in 1986 (6). The genome sequence of a different strain, FDC 92A2, was obtained by TIGR, annotated by the Los Alamos National Laboratory, made publicly available in 2005 (7), and released through the NCBI in 2013 as GenBank accession number CP003191. Regrettably, the genome of strain FDC 92A2 was erroneously attributed to the type strain ATCC 43037 in CP003191. After repeated cases of finding mismatches between the sequence of amplified PCR products from ATCC 43037 and the CP003191 genome, we decided to perform shotgun sequencing of ATCC 43037 and discovered the strain attribution problem. Recently, *T. forsythia* strain FDC 92A2 was made available through ATCC under the deposit number ATCC BAA-2717, and the strain attribution of CP003191 was corrected at the NCBI as of 10 April 2015.

Here, we present the draft genome of *T. forsythia* strain ATCC 43037. The *T. forsythia* ATCC 43037 genome was sequenced using the MiSeq platform (Illumina, Inc.) with an average sequencing coverage of approximately 190×. Sequencing was performed by Microsynth (Balgach, Switzerland), which produced 2,495,150 paired-end reads (250 bp). The reads were subsequently error corrected and *de novo* assembled using SPAdes version 3.1.0 (8). The total sequence length of the assembly is 3,282,277 bp with an average GC content of 47.1%; 141 contigs with an *N*_50_ of 109,101 bp were obtained, where the longest contig was 492,194 bp. The contigs were annotated using the NCBI Prokaryotic Genome Annotation Pipeline with GeneMarkS+ version 2.9. This predicts a total of 2,753 genes, consisting of 2,491 coding sequences, 210 pseudogenes, 44 tRNAs, 7 rRNAs, and 1 noncoding RNA (ncRNA).

### Nucleotide sequence accession numbers.

This whole-genome shotgun project has been deposited at DDBJ/EMBL/GenBank under the accession number JUET00000000. The version described in this paper is the first version, JUET01000000. The genome sequence has also been provided to the Human Oral Microbiome Database (http://www.homd.org).
